# Extended distally based DMCA flap in combination with autologous amputate skin transplantation as a salvage procedure for ring avulsion injury

**DOI:** 10.1007/s00238-015-1164-4

**Published:** 2015-11-14

**Authors:** Till Wagner, Nicholas Slater, Dietmar Ulrich

**Affiliations:** Department of Plastic and Reconstructive Surgery, Radboud University Hospital, Nijmegen, The Netherlands

**Keywords:** Ring avulsion, Finger amputation, DMCA flap, Finger reconstruction

## Abstract

A 23-year-old male student presented to our clinic with a traumatic complex ring avulsion of his right dominant index finger. Clinical evaluation revealed a complete distal amputation of the DIP joint with a laceration of the soft tissue at the middle phalanx and a rupture of the FDP-2-tendon far proximally. We hereby present the patient’s clinical outcome after reconstruction with a distally based extended DMCA-II flap. To our own knowledge, this is the first report of an extended distally based DMCA flap for coverage of a class IVd ring avulsion injury in combination with autologous amputate skin transplantation.

Level of Evidence: Level V, therapeutic study.

## Introduction

Ring avulsion injuries are difficult to treat despite more and more upcoming indications for microsurgical replantations. When amputates are either missing or in poor condition there are usually two options: First, further amputation or ray resection or second, free transplantation. In cases in which the patient isn’t suitable for both, the options are limited. Since its first description, the distally based DMCA flap and its modifications have a broad spectrum of indications. To our knowledge, this is the first described case of a DMCA wraparound modification for finger amputations. In selected cases, this procedure may be a helpful additional reconstructive option.

**Fig. 1:**
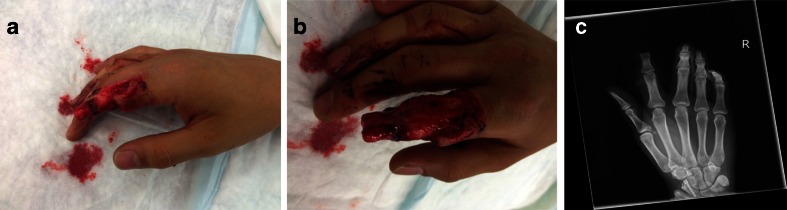
**a** and **b** Preoperative views, **c** Preoperative X-ray of the right hand

## Case report

**Fig. 2:**
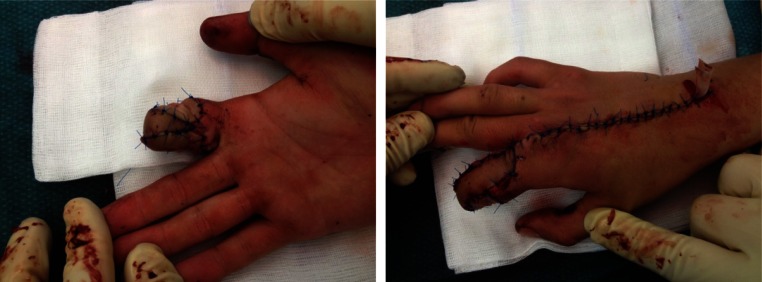
Immediate postoperative result

**Fig. 3:**
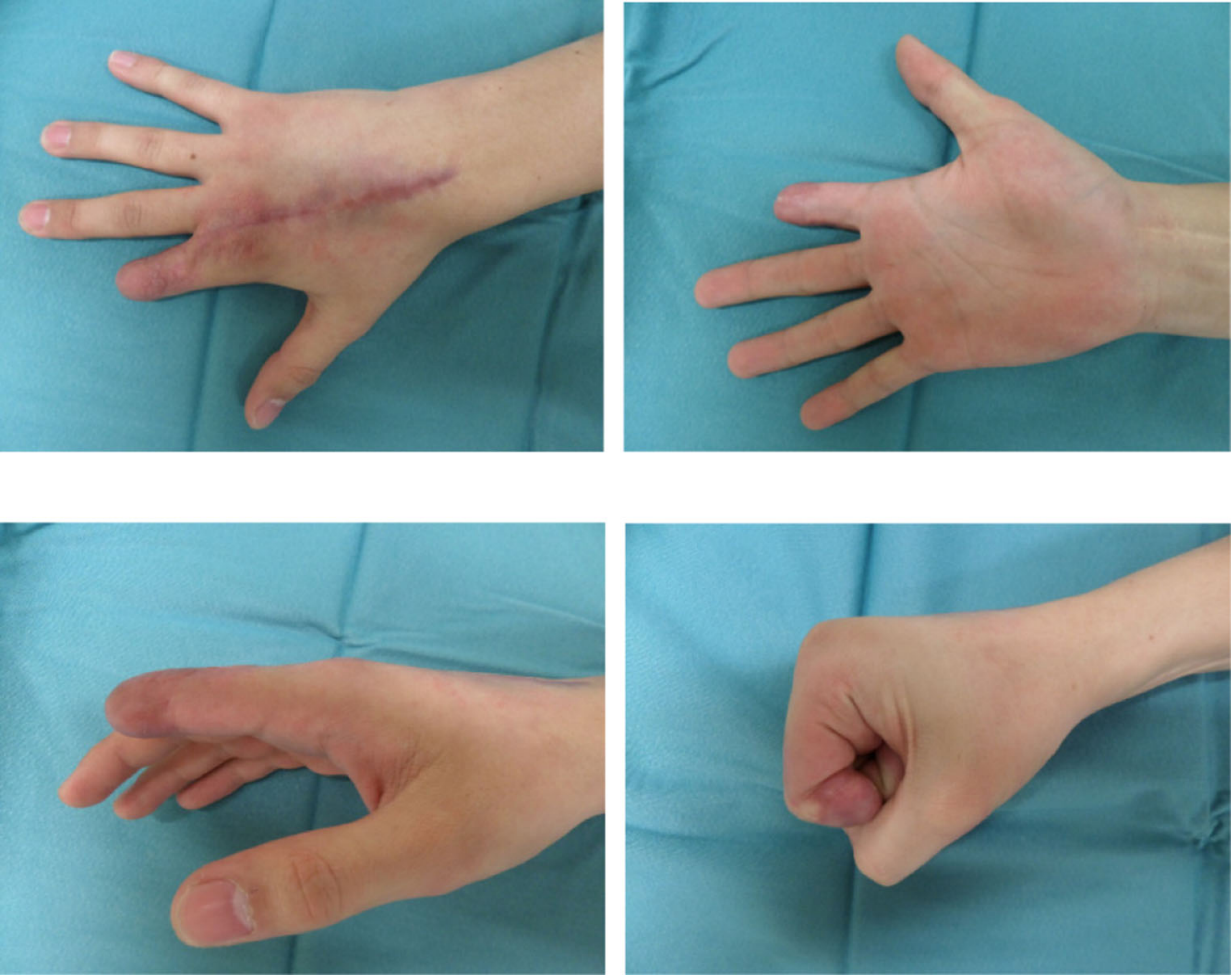
Postoperative view at 6 months with good functional results

On arrival at the emergency department, we saw an adolescent male student with a complete ring avulsion of his right dominant index finger as a result of an unfortunate climb and jump over a bar fence under the influence of energy drink and alcohol. At first, the patient did not recognize his trauma and continued walking after passing the fence, only to return after a short time when he became aware of the situation and collected his amputate and called the ambulance that took him to our hospital (Fig. 1). During transport, the amputate was directly packed in Ringer’s lactate for over 1 h which caused maceration and edema of the soft tissue especially of the vessels. Therefore, replantation was not possible and the patient demanded a local option to keep as much length as possible with a good cosmetic and functional result. Intraoperatively, after exploration and debridement of the amputate, we decided to close the defect with an extended DMCA-II-flap. After drawing the flap design with the support of a template of the defect, we decided to take a slightly different approach with primarily closeable skin at the donor site of the flap and using “spare part” full-thickness skin grafts from the amputate for transplantation (Fig. 2). A volar splint was applied postoperatively for immobilization. The drain was removed after 2 days, and after 3 days, a slight superficial epitheliolysis was observed and treated conservatively. Physiotherapy was started 2 weeks postoperatively. The further clinical course was uneventful. The student returned to his normal life about 3 weeks after the trauma. At his check-up appointment 6 months later, the patient was satisfied with the achieved result. The range of motion in the metacarpophalangeal (MCP) and proximal interphalangeal (PIP) joints were 0-0-90 and 0-0-90, respectively. The active range of motion in his right wrist was not impaired. Blood perfusion had returned to the normal level. Even sensibility was up to the PIP level rated as normal, whereas a more distal two-point discrimination was around 8 mm (Fig. 3).

## Discussion

Since the first description of the DMCA flap in 1990 [1] and his modification, the flap quickly became part of the plastic surgeon’s armamentarium for the coverage of soft tissue defects of the hand [2]. Despite its easy use, the flap has not gained its position in amputation and deglovement injuries. Today, with the ongoing discussion regarding indications for replantation after ring avulsions [3], there is still a lack of surgical options due to absent amputates or amputates in a poor condition. Even in recent studies [4] which have shown more satisfying results in replantation after complete ring avulsion, these issues are not addressed. Most authors agree that amputations distal of the FDS insertion with intact PIP joint are potential candidates for replantation [5]. When the injured amputate is not available or replantable, ray resection is estimated to be the best option except in patients with manual jobs where preserving the MCP joint is said to be favorable [6]. In the current case, we show that a well-known locoregional flap such as the DMCA flap may well serve as a solution where the amputate was not suitable for replantation and the patient negotiates further amputation. The advantage of this procedure is the relatively short operating time with less need for extensive vascular debridement and a short recovery time to normal life. To our knowledge, this is the first description of a case report of a DMCA flap in combination with an autograft from the amputate for a soft-tissue coverage. The patient achieved a good functional and cosmetic outcome and was satisfied with the daily usability of the finger.
